# Draft Genome Sequence of the Aromatic Hydrocarbon-Degrading Bacterium *Sphingobium* sp. Strain Ant17, Isolated from Antarctic Soil

**DOI:** 10.1128/genomeA.00212-14

**Published:** 2014-04-10

**Authors:** Evelien M. Adriaenssens, Leandro D. Guerrero, Thulani P. Makhalanyane, Jackie M. Aislabie, Don A. Cowan

**Affiliations:** a Centre for Microbial Ecology and Genomics, University of Pretoria, Pretoria, South Africa; b Landcare Research, Hamilton, New Zealand

## Abstract

Here, we present the draft genome sequence of *Sphingobium* sp. strain Ant17, an aromatic hydrocarbon-degrading bacterium that was isolated from Antarctic oil-contaminated soil. An analysis of this genome can lead to insights into the mechanisms of xenobiotic degradation processes at low temperatures and potentially aid in bioremediation applications.

## GENOME ANNOUNCEMENT

The ice-free areas of the Antarctic continent, particularly those regions heavily impacted by scientific and/or touristic activities, are less pristine than is generally imagined, with contamination potentially sourced from ground and aerial vehicles associated with research and exploration (1, 2). Bioremediation using microorganisms has been proposed as a potential mechanism for rehabilitating soils impacted by human activities (3). Because of the Antarctic Treaty preventing the introduction of foreign organisms, bacteria with the capacity to degrade aromatic hydrocarbons have been isolated from local contaminated soil (4, 5). *Sphingobium* sp. strain Ant17 was isolated from soil collected near Scott Base on Ross Island, Antarctica, and characterized as an aerobic Gram-negative motile rod able to grow on phenanthrene and 1-methylnaphthalene (5, 6).

*Sphingobium* sp. Ant17 cells were grown on Reasoner’s 2A (R2A) medium at 15°C. DNA was extracted using a combination of bead beating and chemical lysis modified from the method of Miller and colleagues (7). The Ant17 genome was sequenced on an Ion Torrent PGM sequencer (318 Chip) with 400-bp chemistry by the PGM facility of the University of Pretoria. After quality-control filtering and trimming using in-house scripts, 2,268,858 reads were assembled with SeqMan NGen (DNAStar, Madison, WI, USA). The resulting contigs were subsequently merged and cleaned in SeqMan Pro (DNAStar), yielding 199 contigs of >1,500 bp, with a coverage of >20×. The total draft genome length is 5,238,558 bp, with a G+C content of 62%.

Annotation of the contigs was performed using the NCBI Prokaryotic Genome Annotation Pipeline (http://www.ncbi.nlm.nih.gov/genome/annotation_prok), RAST, Aragorn, and RNAmmer (8–10), with RAST identifying 5,291 protein-coding genes, of which 3,354 were not classified in a known subsystem and 1,910 were designated as encoding hypothetical proteins. Sixty-six tRNA genes and three rRNA operons were found, and there are possibly more, since the coverage of these contigs exceeded the average by 2- or 3-fold. The RAST program indicated *Sphingobium japonicum* UT26S to be the closest neighbor to Ant17, with BLASTn analysis showing homology mostly to chromosome 1 of this strain (11, 12).

Analysis of the RAST annotation output revealed a range of interesting genes/operons present in the Ant17 genome. Many genes related to resistance to antibiotics or toxic compounds were found, including genes for metal efflux pumps (e.g., for Cu, Cd, Hg, and As) and multidrug resistance systems, some of which might be located on a plasmid. Several aromatic compound-degrading enzymes were also predicted, although no phenanthrene degradation pathway was identified and the naphthalene-related pathways seemed incomplete. There remains a possibility, therefore, that Ant17 contains the genes for a novel, hitherto unsuspected aromatic compound degradation pathway. Osmotic and oxidative stress-related genes were abundant in the genome, but only one known cold shock protein was identified. This is in accordance with the phenotypic analysis that Ant17 is psychrotolerant (5).

The Ant17 genome analysis reveals possible applications of this organism in the bioremediation of contaminated soil, both in Antarctica and in mesophilic environments.

### Nucleotide sequence accession numbers.

This whole-genome shotgun project has been deposited in DDBJ/ENA/GenBank under the accession no. JEMV00000000. The version described in this paper is the first version, JEMV01000000.
